# Questionable utility of digoxin in left-ventricular assist device recipients: A multicenter, retrospective analysis

**DOI:** 10.1371/journal.pone.0225628

**Published:** 2019-11-25

**Authors:** Mustafa M. Ahmed, Henri Roukoz, Jaimin R. Trivedi, Adarsh Bhan, Ashwin Ravichandran, Rahul Dhawan, Jennifer Cowger, Geetha Bhat, Emma J. Birks, Mark S. Slaughter, Rakesh Gopinathannair

**Affiliations:** 1 Division of Cardiovascular Medicine, Department of Medicine, University of Florida, Gainesville, FL, United States of America; 2 Cardiovascular Division, Electrophysiology Section, University of Minnesota, Minneapolis, MN, United States of America; 3 Department of Cardiovascular and Thoracic Surgery, University of Louisville, Louisville, KY, United States of America; 4 Heart Institute, Advocate Christ Medical Center, Oak Lawn, IL, United States of America; 5 St. Vincent Heart Center of Indiana, Indianapolis, IN, United States of America; 6 University of Nebraska, Omaha, Nebraska, United States of America; 7 Henry Ford Hospital, Detroit, Michigan, United States of America; 8 Division of Cardiovascular Medicine, University of Louisville, Louisville, Kentucky, United States of America; 9 Kansas City Heart Rhythm Institute and Research Foundation, Overland Park, Kansas, United States of America; Thomas Jefferson University, UNITED STATES

## Abstract

**Background:**

While clinical experience with left ventricular assist devices (LVAD) continues to grow and evolve, little is known regarding the ongoing use of certain medications in this population. We sought to evaluate the utility of digoxin in LVAD recipients and its association with outcomes.

**Methods:**

A total of 505 patients who underwent continuous-flow LVAD implantation at 5 centers from 2007–2015 were included. Patients were divided into 4 groups: not on digoxin at any time (ND; n = 257), received digoxin pre implant (PreD; n = 144), received digoxin pre and post implant (ContD; n = 55), and received digoxin only post implant (PostD; n = 49). Survival and all-cause readmission were compared between the 4 groups.

**Results:**

There was no difference in survival at 1 year nor at 3 years between groups (ND = 88%, 66%, respectively; PreD = 85%, 66%; ContD = 86%, 57%; PostD = 90%, 51%; p = 0.7). Readmission per 100 days also was not different between groups (ND = 0.5, PreD = 0.6, ContD = 0.5, PostD = 0.7; p = 0.1).

**Conclusions:**

In this large, multicenter cohort, use of digoxin was not associated with any significant benefit in regard to mortality or hospitalization in patients supported with a continuous-flow LVAD. Importantly, its discontinuation post implant did not worsen all-cause hospitalization or survival.

## Introduction

Despite advancements in pharmacologic therapy for chronic heart failure (HF), digoxin remains a routinely utilized medication. In the years after the landmark Digitalis Investigation Group (DIG) trial, digoxin use upon discharge from a HF hospitalization has decreased from more than half of patients to roughly 1 in 4 [1]. This is likely due to both the improved HF armamentarium available to clinicians, and the results of the DIG trial itself demonstrating a lack of survival benefit with a potential signal for harm with regard to arrhythmias [2]. This has led to a de-emphasis of digoxin in the management of HF, with its presumed utility being limited to a potential reduction in HF hospitalizations [3]. This is often restricted to patients with atrial fibrillation, as more recent analyses have suggested worse outcomes in those HF patients who are on a contemporary medical regimen and in sinus rhythm [4].

The utility of digoxin in patients who go on to receive left ventricular assist devices (LVAD) is less clear. Despite limited data, many LVAD patients are continued on digoxin therapy post implant; one recent study utilizing a large commercial insurance database to evaluate medication adherence in this population noted that nearly 20% were prescribed digoxin [5]. Current guidelines for the medical management of LVAD patients endorse the limited use of digoxin for those patients with rapid atrial fibrillation [6].

Although used commonly in clinical practice, and while its use remains within consensus guidelines, the benefit of digoxin in LVAD patients has not been assessed. In this multicenter study, we sought to evaluate the impact of digoxin use on survival and all-cause hospitalization in a continuous-flow LVAD population.

## Methods

A total of 505 patients who received continuous-flow LVADs at 5 centers in the United States (University of Louisville, Louisville, KY; University of Minnesota, Minneapolis, MN; Advocate Christ Medical Center, Oak Lawn, IL; University of Florida, Gainesville, FL; St. Vincent Heart Center, Indianapolis, IN) between 2007 and 2015 were included in this analysis. The University of Louisville served as the data coordinating center, and the protocol was approved by the Institutional Review Board of each participating center which included a waiver for the informed consent

As the focus of this investigation were longer term associations with digoxin utilization, patients who did not survive their implant hospitalization were excluded. All patients were implanted as bridge-to-transplantation (BTT) or as destination therapy (DT) with a HeartMate II (n = 406) or HeartWare (n = 99) device. The study population was divided into 4 groups based on their use of digoxin relative to device implantation as assessed during the implant hospitalization:

1. No Digoxin therapy: ND (n = 257)
2. Digoxin therapy prior to LVAD implantation only: PreD (n = 144)
3. Digoxin therapy prior to LVAD implantation and continued thereafter: ContD (n = 55)
4. Digoxin therapy after LVAD implantation only: PostD (n = 49)

This was a retrospective analysis. Background sociodemographic variables as well as etiology of HF, type of LVAD, indication for implantation, medication use, and comorbid conditions were included. The day of LVAD implant marked the start date for follow-up. The last day of follow-up was August 31, 2016, or date of heart transplantation, LVAD explanation, or date of death, whichever came first. Mortality and all-cause hospitalization were compared between the 4 groups.

The study groups were evaluated using univariate statistical methods. The non-parametric Kruskal-Wallis test was used for continuous variables and chi-square estimates were used for categorical variables to evaluate the baseline characteristics (Table 1). To analyze the readmission information, numbers of readmissions (overall or cardiac) were converted to readmission per 100 days of device support. To adjudicate the presence of any arrhythmias, device interrogations were utilized with ventricular arrhythmia defined as sustained ventricular tachyarrhythmias lasting >30 s or requiring ICD therapy (antitachycardia pacing or shocks). Atrial arrthythmia was defined as atrial tachycardia, atrial flutter, or atrial fibrillation lasting either >6 hours or ≥1% burden on device interrogation or requiring pharmacologic or electrical therapy for termination. Kaplan-Meier survival estimates were used to evaluate overall survival between the study groups, and were compared using the log-rank test. A cox proportional hazard model was also generated using factors with p<0.1 between the study groups in Table 1. The study groups, i.e. digoxin use, was forced in the model as a hazard factor. All the statistical analysis was done using SAS 9.4 software (SAS Inc., Cary, NC) at a 95% confidence level.

**Table 1:** Baseline demographics by groups.

	ND (n = 257)	PreD (n = 144)	ContD (n = 55)	PostD (n = 49)	*P* value
Age	61 (52–67)	60 (51–69)	59 (50–70)	59 (47–65)	0.5
Male Gender	79%	82%	80%	88%	0.5
BMI	29 (24–34)	28 (23–33)	28 (25–33)	29 (25–34)	0.7
Median Duration of Support (days)	486	607	852	538	0.01
NICM	48%	45%	48%	53%	0.8
BTT	50%	45%	51%	39%	0.4
CRT device	52%	58%	44%	35%	0.02
Pre VA	64%	73%	37%	52%	0.0002
Pre AA	38%	47%	44%	49%	0.2
QRS Duration	140 (114–168)	148 (116–172)	135 (108–160)	140 (110–181)	0.3
EF	15 (10–20)	15 (12–20)	15 (10–18)	15 (10–18)	0.2
Pre LVEDD	6.9 (6.3–7.7)	7.1 (6.5–7.8)	7.1 (6.4–8.1)	7.1 (6.7–7.9)	0.1
INTERMACS					
1,2	29%	23%	41%	46%	0.005
3	34%	27%	14%	17%	
4	20%	27%	21%	12%	
5+	17%	23%	24%	25%	
ACEi	35%	44%	47%	38%	0.4
ARB	17%	18%	9%	16%	0.4
BB	82%	92%	85%	77%	0.02
Amiodarone	35%	36%	35%	33%	0.9
Loop Diuretic	84%	91%	96%	94%	0.02
Thiazides	9%	11%	2%	2%	0.06

ACEi, angiotensin converting enzyme inhibitor; ARB, angiotensin receptor blocker; BB, beta-blocker; BMI, body mass index; BTT, bridge-to-transplantation; ContD, received digoxin pre and post implant; CRT, cardiac resynchronization; EF, ejection fraction; INTERMACS, Interagency Registry for Mechanically Assisted Circulatory Support classification at time of implant; ND, not on digoxin at any time; NICM, nonischemic cardiomyopathy; PostD, received digoxin only post implant; Pre AA, pre-implant atrial arrhythmia; PredD, received digoxin pre implant; Pre LVEDD, left ventricular end diastolic dimension in cm prior to implant; Pre VA, pre-implant ventricular arrhythmia; data represented as median (interquartile range) or %.

## Results

A majority of patients (51%) were not on digoxin at any time point during analysis, comprising the ND group. Of those patients who were taking digoxin pre LVAD, 58% had this therapy discontinued after LVAD implantation, comprising 29% of the overall population and defining the PreD group. 11% of the total population was maintained on therapy post implantation, which defined the ContD group, with another 10% initiating digoxin therapy after implantation, defining the PostD group. Baseline demographics and clinical characteristics of each group are shown in Table 1. There were no significant differences in mean age, predominance of male sex, body mass index, implant strategy, or etiology of HF between groups. The groups did differ in their utilization of cardiac resynchronization (CRT), incidence of pre-implant ventricular arrhythmias, or Interagency Registry for Mechanically Assisted Circulatory Support (INTERMACS) classification at time of implant, with more INTERMACS 1 and 2 patients in the ContD and PostD groups.

**Fig 1:**
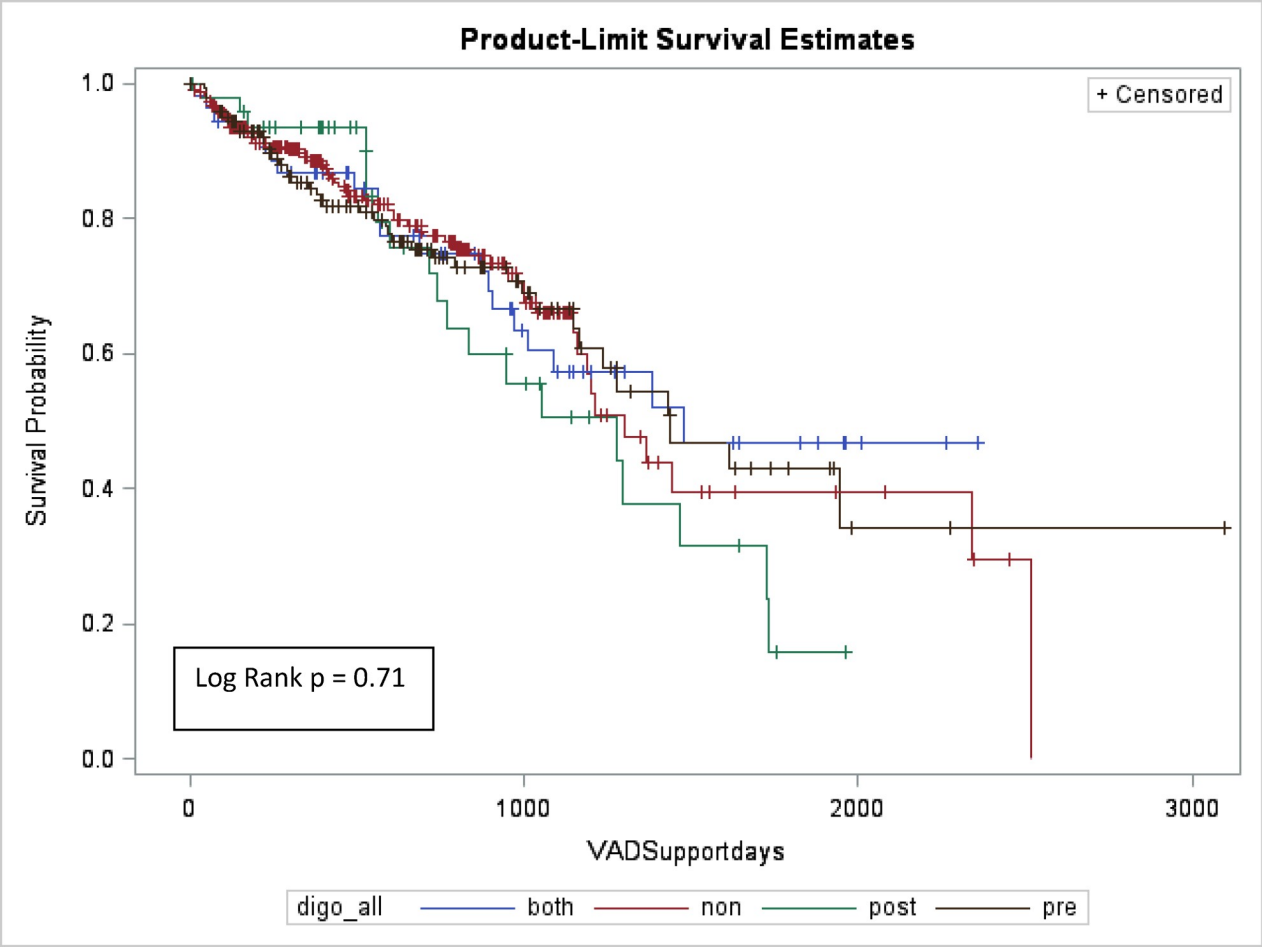
Kaplan Meier survival curves.

When evaluating outcomes in univariate fashion, there were no differences in the incidence of post-implant atrial or ventricular arrhythmias nor in overall survival between groups. Survival at 90 and 180 days, as well as at 1 and 3 years post implant, were also not significantly different (ND = 96%, 93%, 88%, 66%; PreD = 95%, 93%, 85%, 66%; ContD = 94%, 92%, 86%, 57%; PostD = 95%, 93%, 90%, 51%; p = 0.7) (Table 2). All-cause readmission per 100 days was also similar between all 4 groups (ND = 0.5, PreD = 0.6, ContD = 0.5, PostD = 0.7; p = 0.1) (Table 2). Further analysis with cox regression modelling yielded similar results, with no differences in the survival outcomes between groups when evaluating digoxin status, presence of CRT, pre-implant ventricular arrhythmia, pre-implant left ventricular dimensions, or INTERMACS classification (Table 3). As baseline utilization of beta-blockers and loop diuretics varied amongst the groups (Table 1) this too was examined in our cox regression model and demonstrated no survival difference based on medication utilization (Table 3). The same was true when examining those patients who had any exposure to digoxin versus those who had no exposure to digoxin (Table 3). Kaplan-Meier analysis showed no significant difference in survival between the 4 groups at end of follow-up (log rank p = 0.71) (Fig 1).

**Table 2:** Post VAD outcomes and survival by digoxin utilization.

	ND (n = 257)	PreD (n = 144)	ContD (n = 55)	PostD (n = 49)	*P* value
Readmission per 100 days	0.5 (0.2–0.9)	0.6 (0.2–1.1)	0.5 (0.2–1.3)	0.7 (0.4–1.3)	0.1
CV readmission per100 days	0.17 (0–0.42)	0.19 (0–0.50)	0.15 (0–0.30)	0.14 (0–0.46)	0.5
Ventricular arrhythmia	36%	38%	24%	21%	0.1
Atrial arrhythmia	52%	63%	42%	51%	0.07
Survival					0.7
** **90 days	96%	95%	94%	95%	
** **180 days	93%	93%	92%	93%	
** **1 year	88%	85%	86%	90%	
** **3 years	66%	66%	57%	51%	

ContD, received digoxin pre and post implant; CV, cardiovascular; ND, not on digoxin at any time; PostD, received digoxin only post implant; PredD, received digoxin pre implant; VAD, ventricular assist device.

**Table 3:** Cox regression model for all cause mortality.

Variable	Hazard Ratio	*P* value
CRT	1.03	0.9082
Pre VA	1.21	0.4494
ContD	1.35	0.3957
PostD	1.10	0.8109
PreD	1.06	0.8249
AnyD	1.05	0.8394
BB	0.81	0.4998
Loop Diuretic	1.52	0.2703
Pre LVEDD	0.72	0.0084
INTERMACS Classification	1.12	0.1311

AnyD, received digoxin at any time; BB, beta-blokcer; ContD, received digoxin pre and post implant; CRT, cardiac resynchronization; Pre VA, pre-implant ventricular arrhythmia; PostD, received digoxin only post implant; PredD, received digoxin pre implant; Pre LVEDD, left ventricular end diastolic dimension in cm prior to implant; INTERMACS, Interagency Registry for Mechanically Assisted Circulatory Support classification at time of implant.

## Discussion

To our knowledge, this is the first multi-center description of digoxin use in an LVAD population. That its utilization would not result in a survival benefit is not unexpected given its lack of a survival benefit in chronic HF patients. This lack of benefit would likely be magnified in an LVAD population, particularly as early investigation demonstrated blunted hemodynamic effects of digoxin in those HF patients who had normalized hemodynamics with medical therapy [7, 8]. One would expect the same lack of benefit in a mechanically unloaded heart. Furthermore, in addition to the previously mentioned findings of the DIG trial, a retrospective, single-center analysis of patients with advanced HF referred for transplant evaluation also demonstrated no benefit. Importantly, these patients were on a contemporary regimen of HF management, with more than 90% being maintained on both beta-blockers and an angiotensin-converting enzyme inhibitor or angiotensin receptor blocker, with more than 70% having a cardiovascular implantable electronic device [9].

Of more interest is the finding that cessation of digoxin in the PreD group did not result in worsened outcomes, nor did the ContD group have improved outcomes. This is in contrast to early data suggesting that withdrawal of digoxin in chronic HF patients resulted in an increased incidence of ‘treatment failure’, a composite of increased diuretic therapy, emergency-department treatment, HF admission, and adverse events [10]. The finding of clinical deterioration after digoxin withdrawal was also noted in subsequent investigations [11]. This was followed by further analysis of the DIG trial data demonstrating that discontinuation of digoxin was associated with a significant increase in all-cause hospitalization as well as HF hospitalization, while the continuation of digoxin at low serum concentrations resulted in a reduction in all-cause mortality [12]. Although these trials were done prior to the advent of contemporary HF medication regimens, they raised the possibility that stopping digoxin could be harmful even in a present-day HF population. This may explain the continued utilization of this medication seen in 11% of our study population and nearly 20% of those LVAD patients in a private insurance database [5]. Our findings would suggest that stopping digoxin is not harmful, and given the lack of benefit seen with its continuation, cessation of therapy may be considered in selected patients.

The focus on cessation of medical therapies with questionable benefit in the LVAD population should be one of intense interest, as these patients are often on numerous agents and considering the known risk that polypharmacy carries within the HF population [13]. Additionally, LVAD patients have been demonstrated to have variable medication adherence, and therefore attention to simplifying their regimen would be of importance [5]. Complicating this further is the highly variable pricing of digoxin and, as one recent multi-state analysis demonstrated, that it is the most expensive agent of a generic HF regimen [14]. Finally, digoxin is known to have many drug-drug interactions, including carvedilol and loop diuretics, has a relatively narrow therapeutic range, and can result in significant gastrointestinal and central nervous system adverse effects [15] Therefore, the discontinuation of digoxin in selected patients appears attractive.

Despite these concerns, however, there may be certain circumstances in which digoxin therapy in LVAD patients is indicated. That 10% of patients fell into the PostD group would suggest that digoxin may have been used for worsening atrial fibrillation, or perhaps more likely, as an adjuvant therapy for myocardial recovery post implantation. The use of digoxin as part of a pharmacologic protocol to aid in left ventricular recovery after LVAD implantation has been described and may be of utility to achieve improvement in myocardial function in a maximally reverse remodeled heart [16]. Beyond its potential benefit in a protocolized approach to pharmacologic therapy and monitoring for recovery, it is possible that some centers would utilize digoxin post LVAD implantation to aid with right ventricular function. While not studied nor described in this population, data exist that would suggest an improvement in right ventricular function with short-term intravenous administration of digoxin as well as long-term oral therapy in patients with pulmonary hypertension [17, 18]. Indeed, in our analysis, there was a greater proportion of INTERMACS profile 1 and 2 patients in the PostD and ContD groups without a noted difference in survival despite their increased baseline level of illness. This may suggest an efficacy in maintaining or improving right ventricular function and may explain the preferential utilization of digoxin in these patients. As both acute right ventricular dysfunction after LVAD implantation and late right ventricular dysfunction and failure continue to be challenging scenarios, further study into the potential benefit of digoxin may be warranted in this sub-population.

An additional circumstance in which digoxin utilization in LVAD patients may be those who have angiodysplasia-related gastrointestinal bleeding. This was recently described in a single center, retrospective analysis wherein digoxin use was associated with a reduction in angiodysplasia-related gastrointestinal bleeding [19]. Although our data collection pre-dates the publication of this analysis, it is unclear if the proposed mechanism of digoxin’s effect on neoangiogenesis, reduction in the stimulation of angiopoeietin-2 via inhibition of hypoxia-induced factor 1α, may have influenced providers in starting digoxin in the PostD group. The data reported by Vukelic et al did not include an analysis of readmissions, and unfortunately our data does not include an analysis of the incidence of gastrointestinal bleeding making comparison difficult. It can, however, be argued that in this larger, multi-center study, the lack of any difference in readmissions is suggestive of a neutral effect on bleeding as it is a leading cause of both 30 day post implant readmissions as well as longer term hospitalizations [20, 21]. Prior single-center, retrospective studies have discovered other potentially attractive medications for the reduction in angiodysplasia-related gastrointestinal bleeding in the LVAD population, such as inhibition of angiotensin II [22]. The more recent data from Vukelic et al does not endorse this prior finding, again suggestive of the limitations of single center data and the highly complex and multifactorial nature of bleeding in the LVAD population. Alternative explanations for the apparent discordance between our data and that of Vukelic et al would include the lack of digoxin levels in our data while that was obtained in the majority of patients in the latter analysis. The role of digoxin, as well as other potential inhibitors of neoangiogenesis, certainly warrants prospective and randomized investigation.

### Limitations

Our study has several limitations, chief amongst them being its retrospective nature. While the multicenter experience and relatively large numbers help to overcome this issue to some extent, that there was no pre-specified LVAD medical management protocol is also a limitation. Additionally, as a multi-center analysis, pre implant management and selection criteria may have influenced the outcomes. There was no uniform assessment of right ventricular function post implant, so we are not able to comment on use of digoxin specifically for the treatment of right ventricular failure. Furthermore, digoxin utilization was based on abstraction from the medical record without assessment of dosing strategy nor digoxin levels. Future investigation with strict adherence to a post-implantation medication and management protocol would be helpful in strengthening these results. Beyond digoxin levels, the lack of having precise knowledge of the dose of other medical therapies, as well as any dosing titrations during the period of inquiry is a potential confounder. Additionally, the lack of gastrointestinal bleeding data limits our ability to comment on a potentially novel indication for the use of digoxin in the LVAD population.

## Conclusions

In this large, multicenter analysis, digoxin use post continuous-flow LVAD implantation did not have an effect on mortality nor all-cause hospitalization, suggesting that it is of questionable benefit in this population. Given that it may be harmful in certain patients, and that it can increase the complexity of the LVAD patient’s medical regimen at a variable expense, we would argue that digoxin should not be routinely utilized post LVAD implantation. Its potential benefit in selected patients as part of a myocardial recovery protocol, to aid in right ventricular function, or reduce the incidence of angiodysplasia-related gastrointestinal bleeding requires more investigation in a prospective and randomized fashion.

## Supporting information

S1 File EPVAD_DIG_died. Identified raw data set utilized for all analyses.
(XLSX)
